# Development of in situ crosslinked hyaluronan as an adjunct to vitrectomy surgery

**DOI:** 10.1007/s10856-023-06757-9

**Published:** 2023-11-06

**Authors:** Kiyoshi Suzuki, Ippei Watanabe, Takashi Tachibana, Kenichiro Mori, Keijiro Ishikawa, Tatsuro Ishibashi, Eiichi Uchio, Koh-Hei Sonoda, Toshio Hisatomi

**Affiliations:** 1 Central Research Laboratories, Seikagaku Corporation, 1253, Tateno 3-chome, Higashiyamato-shi, Tokyo, 207-0021 Japan; 2 Medical Affairs, Seikagaku Corporation, 1-6-1 Marunouchi, Chiyoda-ku, Tokyo, 100-0005 Japan; 3 Department of Ophthalmology, Graduate School of Medical Sciences, Kyushu University, 3-1-1, Maidashi, Higashi-ku, Fukuoka, 812-8582 Japan; 4 Department of Ophthalmology, Faculty of Medicine, Fukuoka University, 8-19-1, Nanakuma, Fukuoka Jonan-ku, Fukuoka, 814-080 Japan; 5 Department of Ophthalmology, Fukuoka University Chikushi Hospital, 1-1-1 Zokumyoin, Chikushino-shi, Fukuoka, 818-8502 Japan

**Keywords:** In situ crosslinked hyaluronan, Ophthalmic viscoelastic device, Removal, Residual vitreous cortex, Vitrectomy

## Abstract

Ophthalmologists have used hyaluronan (HA) products as adjuncts to ocular surgery since the 1970s. However, HA products are not always functional in surgeries of the posterior eye segment due to their lack of biomechanical strength. In this study, we developed an in situ crosslinked HA (XL-HA) and evaluated its potential as an adjunct to vitrectomy surgery in an in vitro model with a triamcinolone acetonide (TA) layer used as a pseudo residual vitreous cortex (RVC). Within a few minutes at concentrations over 0.9%, XL-HA, generated by the click chemistry of HA-dibenzocyclooctyne and HA-azidoethylamine, formed a hydrogel with the appropriate hardness for tweezers peeling. XL-HA (concentration, 0.76–1.73%) without dispersion successfully entered the TA layer and removed more than 45% of the total TA. Dynamic viscoelasticity helps to explain the rheological behavior of hydrogels, and the assessment results for XL-HA indicated that suitable concentrations were between 0.97% and 1.30%. For example, 1.30% XL-HA hydrogel reached sufficient hardness at 3 min for tweezers peeling, and the TA removal ability exceeded 70%. These results demonstrated that XL-HA was a potential adjunct to successful vitrectomy.

**Graphical Abstract:**

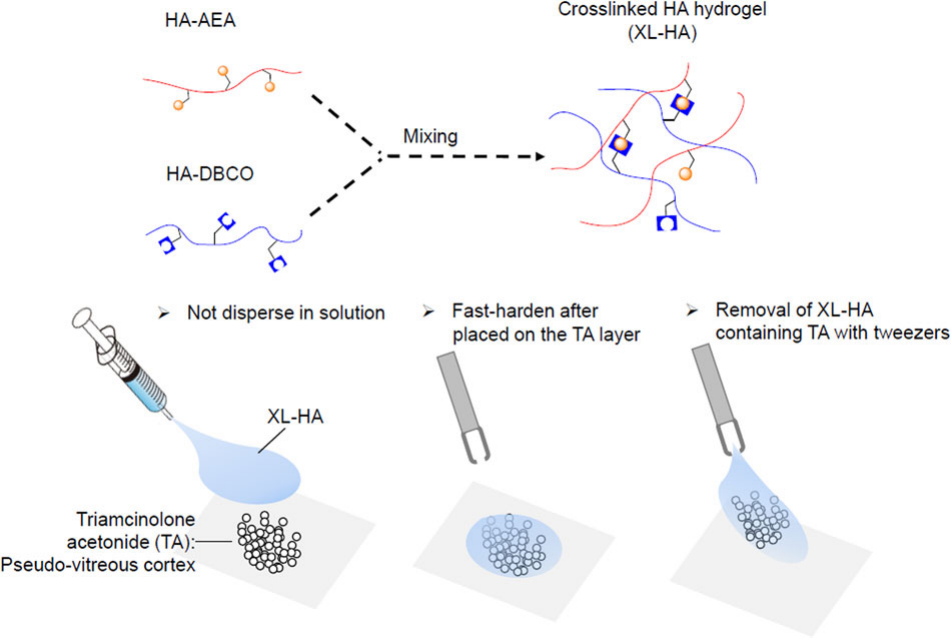

## Introduction

The vitreomacular surface plays an important role in various diseases such as epiretinal membrane, macular hole, retinal detachment, proliferative diabetic retinopathy, and proliferative vitreoretinopathy [1–3]. The vitreomacular pathologies often include pathogenic changes such as liquefaction or shrinkage of the vitreous gel; cellular migration; secretion of cytokines, chemokines, and extracellular matrices; membrane formation; membrane traction in the vitreomacular interface. Under pathological conditions, the residual vitreous cortex (RVC) may cause cellular migration on the retina, serving as a scaffold for the proliferation of resident hyalocytes, glial cells, vascular endothelial cells, or infiltrating monocytes and macrophages. Subsequent secretion of extracellular matrices and inflammatory cytokines can cause secondary vitreoretinal interfacial disorders such as macular epiretinal membrane, retinal traction, and secondary retinal detachment. The vitreoretinal diseases thus affect visual prognosis significantly. Therefore, surgical removal of these RVC or proliferative membranes (PMs) is one of the targeted goals for successful vitrectomy surgeries. In other words, surgical techniques to remove the thin and fragile RVC and fibrous PM are necessary for safe and effective vitrectomy surgery.

Many improvements have been achieved in surgical devices and techniques to assist vitrectomy. A device to protect intraocular forceps with polyvinyl alcohol hydrogel has been developed to prevent retinal damage during physical scraping of the RVC from the retina [4]. However, the risk of retinal damage cannot be eliminated because it is difficult to finely control the force applied. Chromovitrectomy, the procedure to stain living tissues or cells for identifying ocular tissues during the vitrectomy, has been developed and has become popular among vitreoretinal surgeons [5–9]. In particular, visualizing agents such as triamcinolone acetonide (TA) and Brilliant Blue G (BBG) have been popular in many countries [10–15]. These tissue staining agents help visualize injured areas on the retinal surface. However, vitrectomy surgeries are still complex and require skilled surgeons who are highly proficient in the procedures.

Solutions of hyaluronan (HA) with high molecular weight have been used most commonly for cataract surgery as ophthalmic viscoelastic devices (OVDs) since the 1970s [17]. Since then, HA products have contributed to technological innovation in ocular surgery due to their high biocompatibility, optical transparency, and rheological properties such as adhesion, elasticity, and cohesiveness [17–20]. Although off-label in vitrectomy, HA products also have been applied to various vitrectomy surgeries such as chromovitrectomy [21–23], viscodelamination [25], and viscodissection [26]. However, almost none of these techniques became common for various reasons. Actually, some of the existing HA products we tested could hardly be picked up with tweezers because of their weak mechanical strength. Besides, those HA products did not maintain their firmness and gradually spread out in aqueous solution. The following properties are necessary for the application of HA-based materials as an adjunct to vitrectomy surgery: injectability via a 27‑gauge needle, which is commonly used in ophthalmic surgery to reach the affected area, solidification in a few minutes (enough to pick up the hydrogel material with tweezers), RCV removal ability, and no cytotoxicity. However, no materials exhibiting such properties have yet been evaluated.

**Fig. 1:**
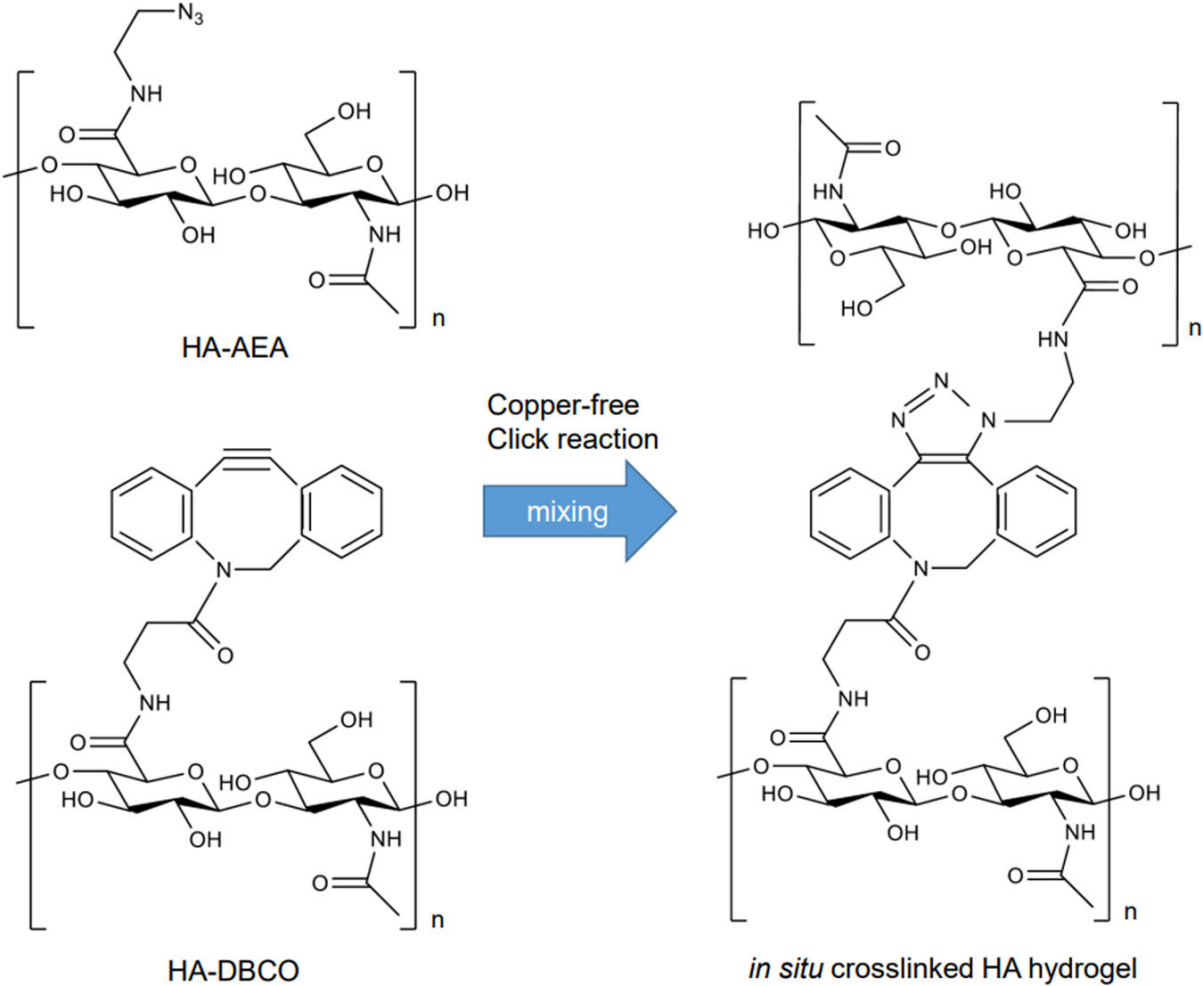
In situ crosslinked hyaluronan hydrogel. Reaction scheme of in situ crosslinked hyaluronan hydrogel (XL-HA)

Crosslinking in polymers forms networks that reinforce polymer mechanical strength [27]. As shown in Fig. 1, in situ crosslinked HA hydrogel (XL-HA) is designed to be prepared by click chemistry [28]. Some XL-HAs have been evaluated as experimental vitreous substitutions [29, 30], and the results of these evaluations indicate that XL-HAs contribute to long-term vitreous stability without retinal toxicity. In this study, we developed a novel cell-free model to assess their efficiency in the stripping of a TA layer serving as a pseudo-RVC and demonstrated that XL-HA has promising potential as an adjunct to vitrectomy.

## Materials and methods

### Materials

HA from *Streptococcus equi* subsp. *zooepidemicus* was obtained from Seikagaku Corp. (Tokyo, Japan). HA with a molecular weight of ~300 kDa (HA300) was prepared by the conventional method [31]. Dibenzocyclooctyne (DBCO)-amine, 4-(4, 6-dimethoxy-1, 3, 5-triazin-2-yl)-4-methyl-morpholinium chloride (DMTMM), 2-chloroethylamine hydrochloride (CEA), and sodium azide were purchased from Tokyo Chemistry Industry (Tokyo, Japan). 2-Azidoethylamine hydrochloride (AEA) was prepared from CEA and sodium azide according to the method described by Willinger and Reimhult [32]. TA, ×100 penicillin-streptomycin (PS) solution, and dimethyl sulfoxide (DMSO) were purchased from Fujifilm Wako Chemicals (Osaka, Japan). BBG and GMX1778, a nicotinamide phosphoribosyl transferase inhibitor, were purchased from Merck KGaA (Darmstadt, Germany). BSS PLUS^®^ 500 Eye Perfusate 0.0184% (BSS) was purchased from Alcon Japan Ltd. (Tokyo, Japan). Fibrin glue (Beriplast^®^ P Combi-Set Tissue Adhesion) was purchased from CSL Behring K.K. (Tokyo, Japan). Beriplast P was prepared according to the manufacturer’s instructions [33]. Solutions A and B in the Beriplast P kit were prepared as follows: Ingredients A (human fibrinogen, 240 mg; human factor XIII, 180 IU; bovine aprotinin, 3000 KIE) and ingredient B (human thrombin, 900 U) were separately dissolved in 3 mL of BSS (undiluted), or each solution diluted tenfold with BSS. To make the hydrogel visible, BBG was added to each solution A at a final concentration of 270 µg/mL. The cohesive OVD, Sodium Hyaluronate Ophthalmic Viscoelastic Preparation 1% “SEIKAGAKU,” hereafter referred to as 1% HA-cohesive, was purchased from Santen Pharmaceutical Co., Ltd. (Osaka, Japan). Gel-One^®^, an XL-HA product containing 1% HA, was purchased from Zimmer Biomet (Warsaw, IN, USA).

### Preparation of HA derivatives, HA-DBCO or HA-AEA as precursors

HA300 was dissolved in 50% ethanol (final concentration, 12.4 mM [5 mg/mL]), mixed with the equivalent of DBCO or AEA in the presence of DMTMM, and stirred at room temperature (20–25 °C) overnight (20–24 h). The reaction was terminated by alkalinization to pH 11–12. Each HA derivative was harvested by conventional ethanol precipitation and stored in a refrigerator until use.

### Determination of the degree of substitution in HA derivatives

1H NMR spectra were recorded on a 500 MHz NMR apparatus (Bruker AVANCE III 500; Bruker Japan, Tokyo, Japan). To assess the degree of substitution (DS) of HA-AEA, it was converted to HA-AEA/DBCO (modified HA-AEA) by reaction with a sufficient amount of DBCO-amine. Each sample was dissolved in 0.7 mL of D_2_O. The degree of substitution (DS) of functional moiety in HA-DBCO or the modified HA-AEA was defined as the number of DBCO or AEA-DBCO moieties per HA disaccharide unit. Based on the 1H NMR spectra, DS (mol/mol) was determined as a ratio of integral values between aromatic protons around 7–8 ppm and N-acetyl methyl protons at 1.9–2.1 ppm of HA-DBCO or the modified HA-AEA [34, 35].

### Preparation of in situ crosslinked HA hydrogels (XL-HA)

Each HA derivative was reconstituted in BSS to give a 1% solution for the standard reaction. Solution HA-DBCO included 520 µg/mL of BBG as a visualizing agent. Both HA derivative solutions (300 µL each) were mixed. After being vortexed for 10 s, the mixture was loaded into a 1 mL syringe with a 27-gauge needle and used immediately for various examinations.

### Measurement of dynamic viscoelastic properties of hydrogels

The dynamic viscoelastic properties, including storage modulus (G’) and loss modulus (G”), of the testing materials (280 µL) were measured simultaneously with a modular compact rheometer, model MCR302 (Anton Paar Japan K.K., Tokyo, Japan) equipped with dedicated probe (model PPT25-SN38699, d = 0.5 mm) under the following conditions: temperature, 25 °C; frequency, 1 Hz; strain amount, 0.1%; measurement interval, 1 sec. The measurement was started immediately after loading the sample and moving the probe to a height of 0.5 mm. The solidity index (S-index: min) was defined as the time when the G’ value of testing materials exceeded 30 Pa for the first time, respectively. Thirty Pa was the minimum G’ value that we could peel off XL-HA hydrogels with tweezers. Furthermore, the liquidity index (L-index: min) was defined as a time when the G”/G’ ratio of testing materials became less than 1 for the first time. All analyses were done with the aid of the computer program RHEOPLUS/32 V3.62 (Anton Paar Japan K.K., Tokyo, Japan). The results are shown as mean values of two measurements.

### In vitro pseudo-RVC models using a TA layer

We developed a clinically relevant in vitro pseudo-RVC model using TA. A 10 mg/mL TA suspension was prepared in BSS. As a qualitative assay, a pseudo-RVC was formed by placing an appropriate amount of TA suspension in the bottom of a 10 cm diameter dish or a 100 mL disposable cup containing 10 ml BSS. Then, testing materials were loaded onto the TA layer for 5 min or 10 min and peeled off with tweezers. As a quantitative assay of TA removal ability, we prepared a TA layer (2 mg/well) on the bottom of each well of a 24-well plate containing 2 mL of BSS. Testing material (350-µL) was loaded onto the TA layer and was removed with tweezers after 5 min or 10 min. Residual TA was collected from each well by centrifugation at 9200 × *g* for 5 min, suspended in distilled water, and the amount of residual TA was determined by measuring turbidity at 660 nm. The measurement was done twice and the data were averaged. The amount of TA recovered (mg) in each well was calculated by using turbidity of a 1 mg/mL TA suspension in BSS as a control. The removal ability was calculated using the following formula: [TA removal, %] = {[Initial TA, 2 mg] – [TA recovered, mg]}/[2 mg] × 100.

### Cytotoxicity assessment of HA derivatives

To assess the cytotoxicity of HA derivatives, the viability of the adult retinal pigment epithelial cell line-19 (ARPE-19, the American Type Culture Collection, Manassas, VA, USA) was determined using the MTT [3-(4,5-dimethylthiazol-2-yl)-2,5-diphenyltetrazolium bromide] assay with the Cell Counting Kit-8 (DOJINDO, Kumamoto, Japan). The base medium for cell proliferation testing was DMEM/F12 with 10% FBS and 1% PS. Cells were seeded at 3.0 × 10^3^ cells into each well of a 96-well plate and incubated at 37 °C, in an atmosphere with 95% humidity and 5% CO_2_ overnight. After changing the medium to one containing different concentrations of HA300, HA-AEA, or HA-DBCO or to the base medium, the culture was continued for 2 days. The absorbance at 450 nm (A_450_) of the culture grown in the base medium was consistently above 2.0. The cell proliferation testing under each condition was done in triplicate. Relative cell proliferation was determined by using the following formula: [Relative cell proliferation, %] = ([A_450_SA] − [A_450_SB]) / ([A_450_CA] − [A_450_CB]) × 100

A_450_SA: A_450_ of the culture containing test substance solution after incubation with MTT,

A_450_SB: A_450_ of the culture containing test substance solution before incubation with MTT,

A_450_CA: A_450_ of the negative control culture after incubation with MTT,

A_450_CB: A_450_ of the negative control culture before incubation with MTT.

Criteria for determination of cytotoxicity were as follows: (i) relative cell proliferation declined by more than 30%, and (ii) morphological changes under microscopic observation. In the presence of 5 µg/mL GMX1778 as cytotoxic control, the cells showed spheroidal morphology and a decrease of 11% in relative cell proliferation.

## Results

### Development of an in vitro assay using a TA layer as a pseudo-RVC

**Fig. 2:**
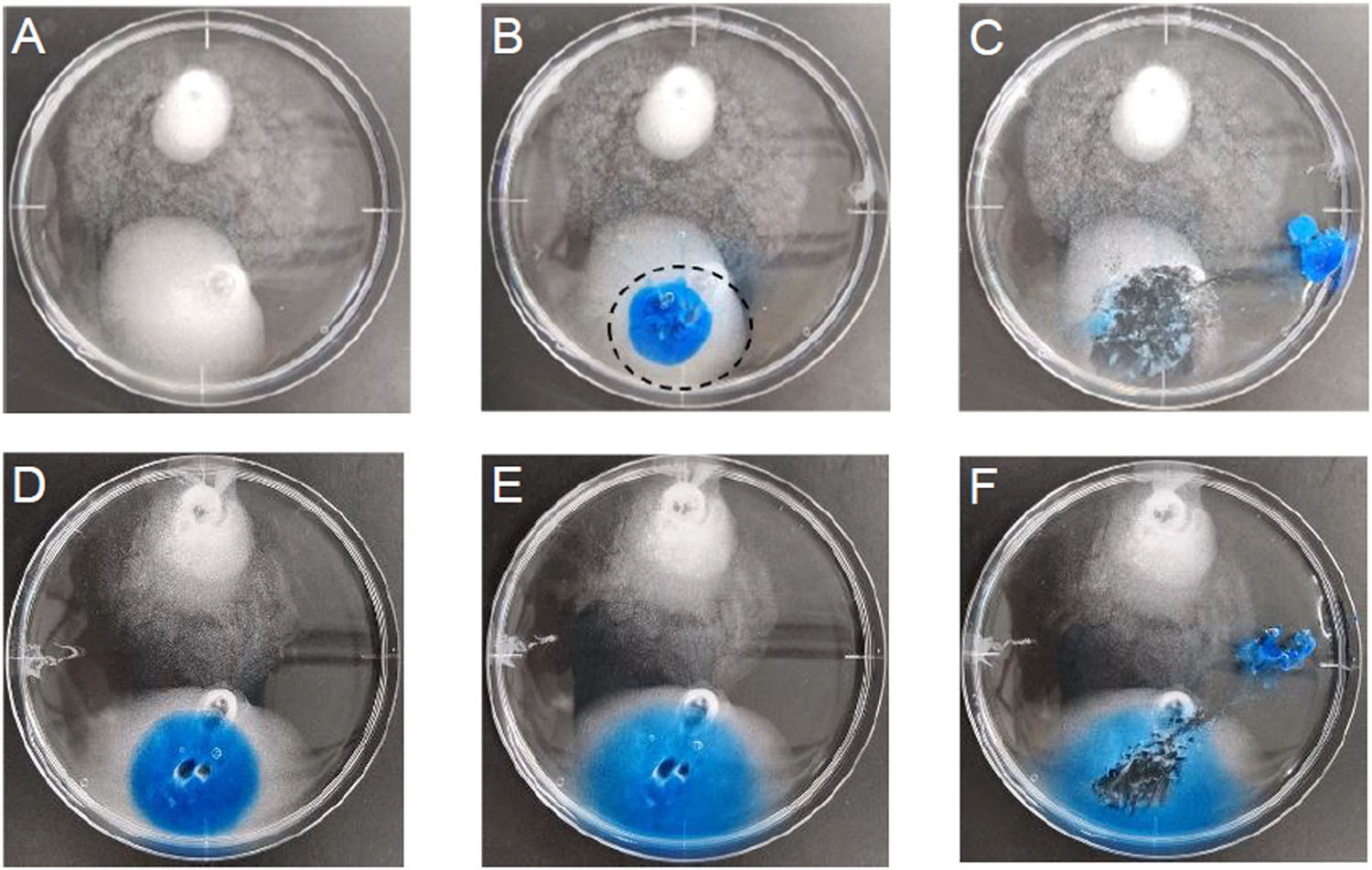
Removal of TA, a pseudo-vitreous cortex, with the Beriplast P. Freeze-dried product A (human fibrinogen, 240 mg; human factor XIII, 180 IU; bovine aprotinin, 3000 KIE) and freeze-dried product B (human thrombin, 900 U) were reconstituted in 3 mL of BSS, solution A and B (undiluted), respectively. Besides, BBG was added to solution A to give a final concentration of 270 µg/mL. **A** TA layer. **B** The Beriplast P hydrogel was formed by using the multi-layer procedure recommended in the manufacturer’s instructions. Briefly, solution A (175 µL) was placed on the TA layer, and the same volume of solution B was placed on the solution A layer. **C** After incubating the mixture under ambient conditions for 5 min, the resulting hydrogel was removed with tweezers. **D**–**F** Beriplast P prepared by mixing diluted solution A and B was also evaluated. **D** Just mixing. **E** After 5 min. **F** Removing Beriplast P

An in vitro assay was developed with TA placed on the bottom of a dish, or a 24-well plate, to explore materials having TA removal ability. The technical practicability of the in vitro assay was examined with Beriplast P (Fig. 2). A multi-layer procedure to separately apply the solutions A and B, and induce spontaneous mixing and hydrogel formation in situ was adopted. Beriplast P prepared by the multi-layer procedure formed a two-part hydrogel that swelled three-dimensionally in BSS: the core containing BBG and a transparent mantle (Fig. 2A–C). Dilution of solutions A and B suppressed the undesirable swelling of hydrogel (Fig. 2D–F). These hydrogels were somewhat ductile when peeled from the dish, but were highly effective in removing nearly all TA in the covered area. The assay was scaled down to 24-well plates to quantify TA removal. Calculated as % of total TA, TA removal by peeling of Beriplast P was 95.3%, and that by peeling of diluted Beriplast P was 97.6%. These results suggested that the excellent performance of Beriplast P was due to its high adhesiveness. Dilution tenfold allowed the Beriplast P to detach smoothly from the well.

### Assessment of dynamic viscoelasticity, G’, and G”, of hydrogels

**Fig. 3:**
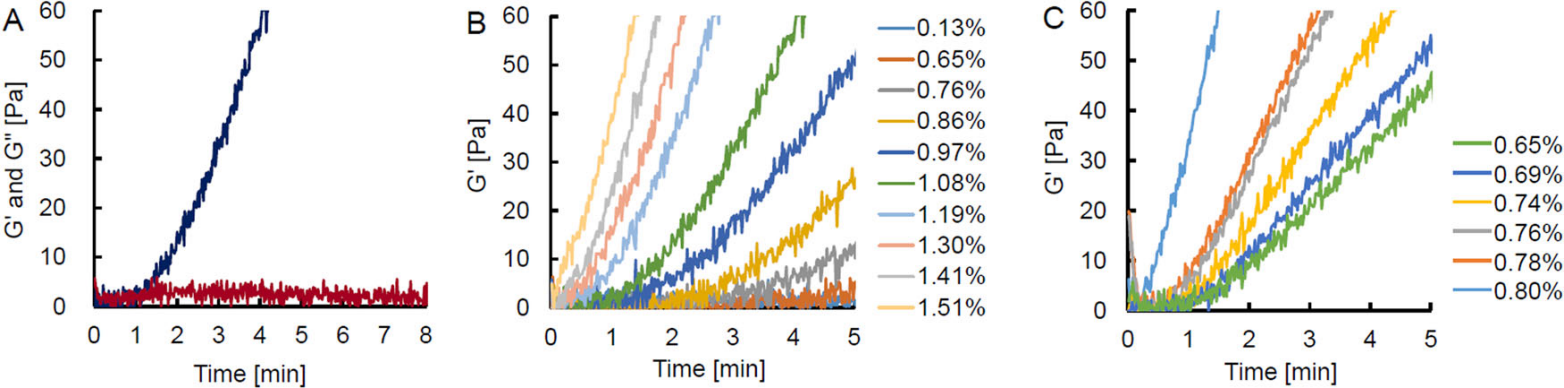
Time-course of dynamic viscoelasticity of various viscoelastic materials. **A** XL-HA: The same volume (140 µL) of 1% solutions of HA-DBCO and HA-AEA prepared as batch 1 were mixed and put on a measuring disc. Dynamic viscoelasticity was measured with a modular compact rheometer: G’, indigo line; G”, maroon line. Effect of concentrations on the dynamic elasticity of XL-HA. (**B**) Time-course of the G’ of XL-HAs prepared from HA derivatives with the DS of 0.09 (batch 1) at different concentrations. **C** Time-course of the G’ of XL-HAs prepared from HA derivatives with the DS of 0.30 (batch 6) at different concentrations

Dynamic viscoelasticity helps to assess the elasticity and viscosity of hydrogels separately. Figure 3A shows a typical time-course in the dynamic viscoelastic properties of 1% XL-HA prepared from HA derivatives with a DS of 0.09. The G’ value of 1% XL-HA increased in a time-dependent manner, and the G” value of 1% XL-HA was a constant value (Average: 2.02 ± 1.22 Pa). The G” value of 1% XL-HA exceeded its G’ value until 1.33 min (L-index) after mixing, indicating that it had liquidity. Subsequently, the G’ value of 1% XL-HA increased exponentially and exceeded 30 Pa after 3.67 min (S-index). The G” value of XL-HA was not affected by concentration and was similar to that of 1% XL-HA (data not shown), whereas the G’ value increased depending on the concentration of the hydrogel (Fig. 3B). Besides, tripling the DS of the HA derivative significantly accelerated the gelation as measured by the G’ value (Fig. 3C).

### Preparation of HA derivatives, HA-AEA and HA-DBCO, as precursors

**Fig. 4:**
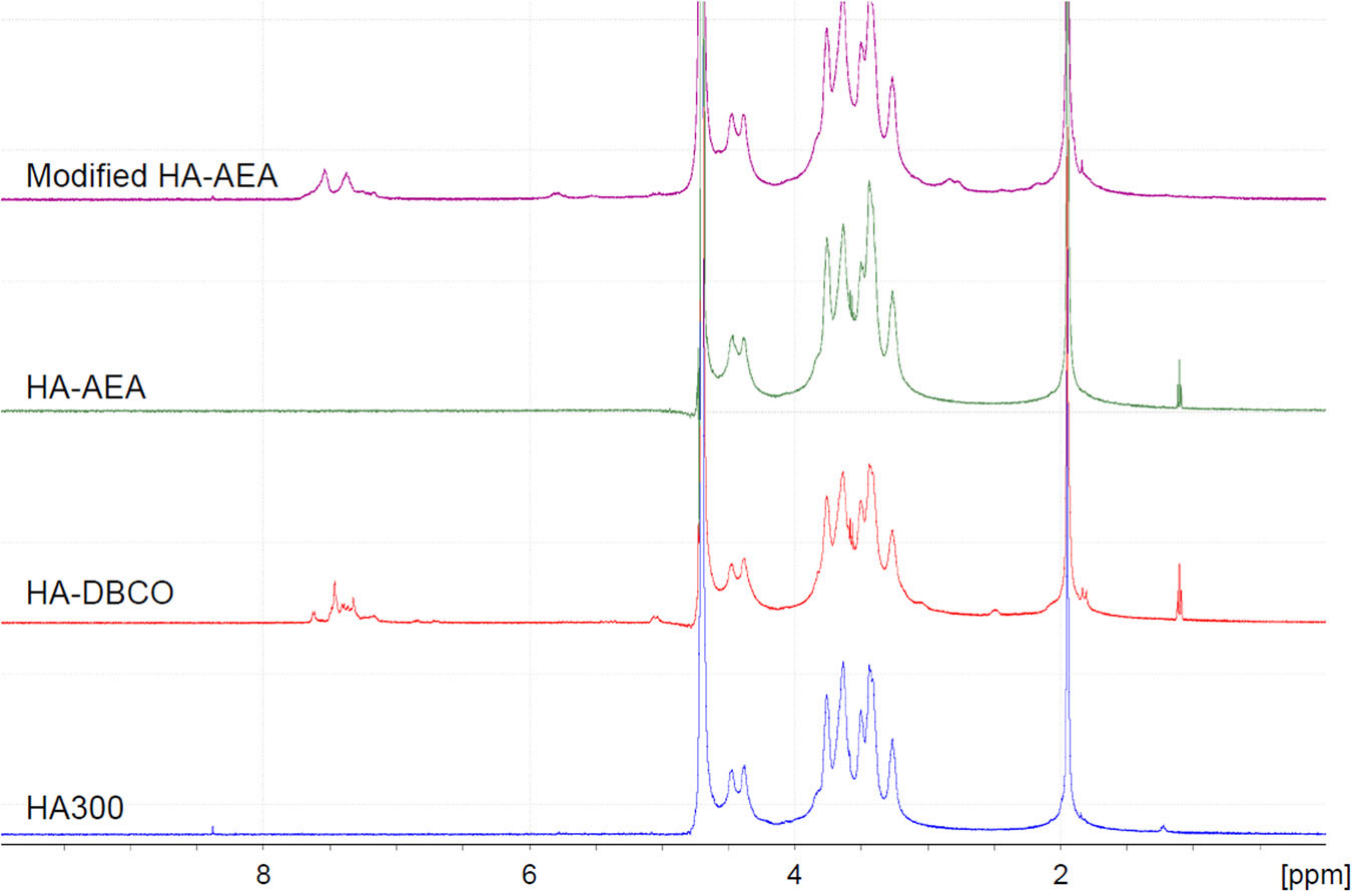
Typical 1H-NMR spectra of HA300, HA-DBCO, HA-AEA, and modified HA-AEA. Modified HA-AEA was prepared by reacting HA-AEA with a sufficient amount of DBCO-amine. HA-AEA, HA-DBCO, and HA300 were evaluated individually. NMR chemical shifts of aromatic protons are observed at around 7–8 ppm and N-acetyl methyl protons are observed at 1.9–2.1 ppm of HA-DBCO or modified HA-AEA

Each HA derivative was prepared in one batch on a 1000 mg scale (batch 1) and 5 batches on a 300 mg scale (batches 2–6) under standard reaction conditions. HA derivatives were also prepared in one batch (batch 6) with threefold amounts of all the reaction agents on the 300 mg scale. Table 1 summarizes the results of all HA derivatives prepared in this study. The weight-based yield of each HA derivative was ~100%. The DS of functional groups in HA derivatives was determined from 1H-NMR spectrum analysis (Fig. 4). The DS of DBCO in each HA-DBCO in batches 1–5 was 0.089–0.092, and that of HA-DBCO in batch 6 was 0.290. The 1H-NMR spectrum of HA-AEA was similar to that of HA300 in appearance, and no signals originating from AEA were found in HA-AEA. To confirm the introduction of the azide group, HA-AEA was modified with DBCO to introduce aromatic rings in the molecule (modified HA-AEA). The modification enabled the determination of the DS of HA-AEA by the same procedure as HA-DBCO: the DS of HA-AEA in batches 1–5 was 0.087–0.103, and that of HA-AEA in batch 6 was 0.300. These results suggested that we could control the DS of functional groups in HA derivatives by changing the amounts of substituting reagents added. To assess the gelation performance of each HA derivative, we determined L- and S-indices of 1% XL-HA prepared by mixing 1% HA-AEA and HA-DBCO solution with a counter HA derivative prepared in batch 1 as the standard. The dynamic viscoelastic properties of HA derivatives prepared in this study were summarized in Table 1. The average values of the L- and S-indices of the 1% XL-HA were 1.43 ± 0.16 min, and 3.78 ± 0.21 min, respectively. Besides, tripling the DS of HA derivatives accelerated gelation drastically.

**Table 1:** Summary of HA derivatives prepared in this study

HA derivative	Batch ID^a^	HA300 (mg)	Mw (kDa)	DS^b^ (mol/mol)	Rheological properties of XL-HA^c^	
					L-index	S-index
					(min)	(min)
HA-DBCO	1	1000	432	0.092	1.33	3.67
	2	300	374	0.090	1.25	3.77
	3		405	0.092	1.27	3.80
	4		435	0.091	1.33	3.85
	5		428	0.089	1.48	4.05
	6		265	0.290	3.27^d^	6.70^d^
HA-AEA	1	1000	259	0.087	1.33	3.67
	2	300	258	0.094	1.38	3.77
	3		262	0.098	1.58	4.03
	4		226	0.103	1.68	3.30
	5		269	0.088	1.63	3.90
	6		241	0.300	0.17^d^	1.27^d^

^a^We prepared HA derivatives under the standard reaction conditions in batches 1–5 and those in batch 6 by adding threefold amounts of DMTMM, DBCO, and AEA

^b^We determined the degree of substitution (DS) by 1H-NMR analysis

^c^For the preparation of 1% XL-HA, each 1% HA derivative prepared in batch 1 was used as the counter standard. Batch 1 of HA-DBCO was used as a standard and mixed with each batch of HA-AEA. Batch 1 of HA-AEA was also used as a standard. Solution HA-DBCO included 520 µg/mL of BBG as a visualizing agent

^d^For the determination of viscoelasticity of HA derivatives in batch 6, 0.78% of HA derivative was used

### In vitro evaluation of TA removal ability of XL-HA

**Fig. 5:**
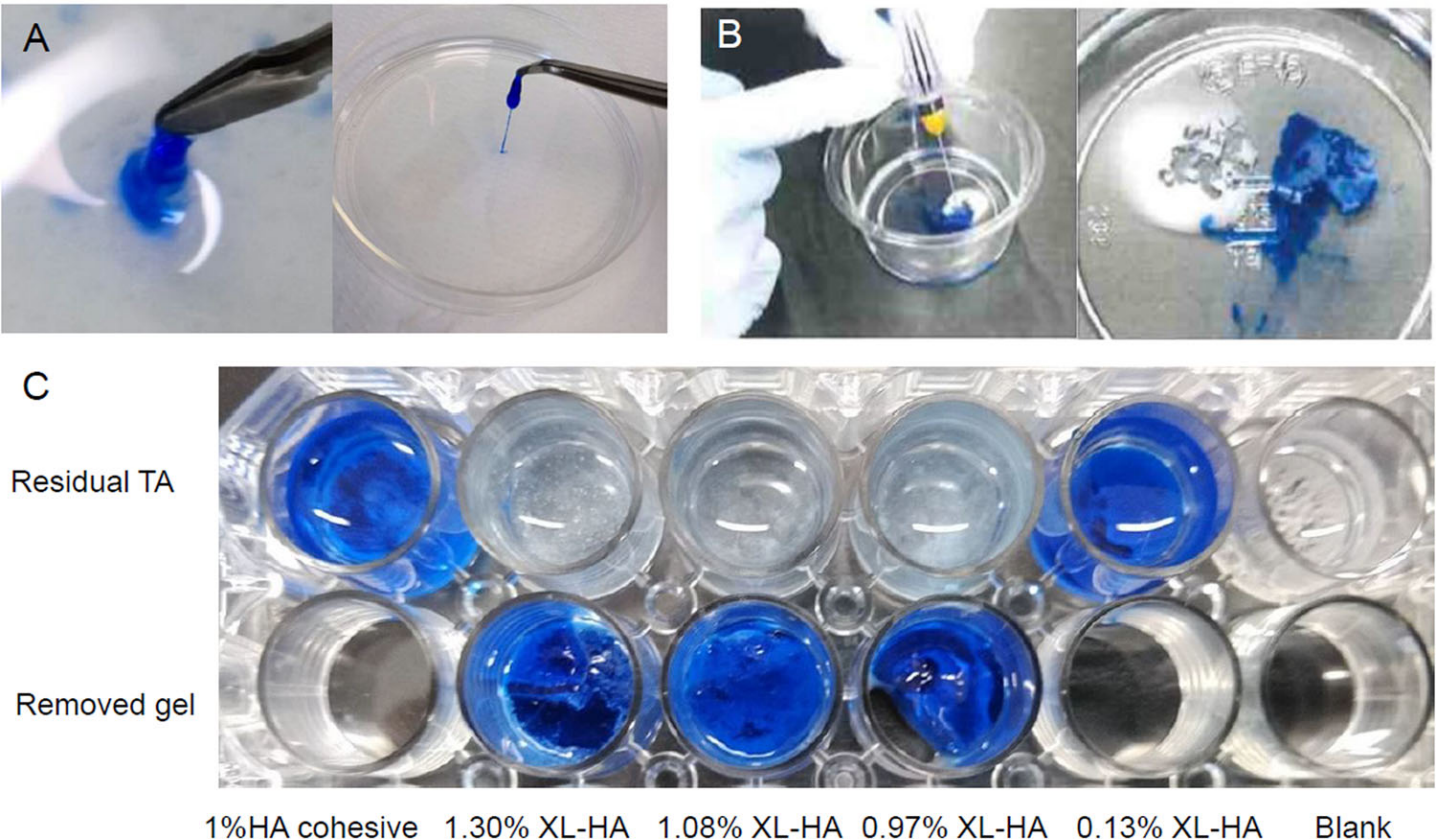
Removal of pseudo-vitreous cortex with the XL-HA. Batch 1 used in this study. **A** Picking up XL-HA after standing in BSS for 3 min. **B** XL-HA was placed on the TA layer (left), and the resulting XL-HA was kept for 5 min and removed with tweezers (right). **C** Quantitative assay using a 24-well plate

As shown in Fig. 5, 1.0% XL-HA passed through a 27-gauge needle smoothly and covered the TA layer. When XL-HA was peeled off after 5 min, TA embedded in XL-HA was also removed. The TA removal ability of different concentrations of XL-HA was assessed qualitatively. TA embedment in 0.13% XL-HA failed due to diffusion in BSS as well as 1% HA-cohesive. TA embedment during hydrogel formation proceeded at XL-HA concentrations ranging from 0.97% to 1.30%, and the TA was successfully removed together when the hydrogel was peeled off after 5 min. Table 2 summarizes the effect of concentration on the characteristics of XL-HA. XL-HA concentration correlated positively with negatively with L- and S-indices, suggesting that the concentration of HA derivatives directly affected hydrogel liquidity and solidity. The L-index of XL-HA at concentrations above 1.51% was 0.0 min, suggesting that the hydrogels could no longer behave as a liquid. At concentrations above 1.62%, the XL-HA showed resistance when discharged through a 27-gauge needle but could be extruded, and the TA removal ability of XL-HA hydrogels was somewhat low. Since XL-HA with concentrations above 1.62% adhered to the wells, their adhesiveness might have contributed to their TA removal ability.

**Table 2:** Effect of XL-HA (batch 1) concentration on the dynamic viscoelastic properties and TA removal ability

Sample	Removal	Solidity		Liquidity		Behavior 5 min after injection into BSS
	Ability^a^ (%)	S-index (min)	Tweezers operation	L-index (min)	Injectability^b^	
1% HA300	0	Not tried	Impossible	Fluid	Easy	Diffusive
1% HA-AEA	0	Not tried	Impossible	Fluid	Easy	Diffusive
1% HA-DBCO	0	Not tried	Impossible	Fluid	Easy	Diffusive
0.13% XL-HA	7	Not tried	Impossible	Fluid	Easy	Diffusive
0.65% XL-HA	Not tried	10.3	Breakable	8.3	Easy	Non-dispersive
0.76% XL-HA^d^	55	–	Stretchable, breakable	–	Easy	–
0.86% XL-HA	45	5.4	Stretchable, breakable	2.8	Easy	Non-dispersive
0.86% XL-HA^d^	84	–	Stretchable, breakable	–	Easy	–
0.97% XL-HA	71	3.8	Easy	1.6	Easy	Non-dispersive
1.08% XL-HA	64	2.9	Easy	1.2	Easy	Non-dispersive
1.19% XL-HA	87	1.8	Easy	1.0	Easy	Non-dispersive
1.30% XL-HA	71	1.6	Easy	0.7	Easy	Non-dispersive
1.41% XL-HA	69	1.2	Easy	0.4	Easy	Non-dispersive
1.51% XL-HA	76	0.8	Easy	0.0	Easy	Thread-shape
1.62% XL-HA	66	0.3	Easy	0.0	Hard	Thread-shape
1.73% XL-HA	59	0.0	Easy	0.0	Hard	Thread-shape
0.78% XL-HA (DS: 0.3)^c^	79	1.2	Breakable	0.0	Easy	Non-dispersive
1% HA-cohesive	0	Not tried	Impossible	0.0	Very hard	Diffusive
Gel-One	0	Not tried	Impossible	0.0	Very hard	Diffusive
Beriplast P; tenfold diluted	97	19	Stretchable	0.2	Easy	Non-dispersive

^a^[TA Removal, %] = {[Initial TA, 2 mg] – [TA recovered, mg]}/[2 mg] × 100. Removal ability was measured in each sample three times and the average value was recorded

^b^Injectability was qualitatively assessed using a 1 ml syringe with an attached 27-gauge needle

^c^HA derivatives prepared as batch 6 (DS: 0.3) were used for the preparation of 0.78% XL-HA

^d^Properties assessed 10 min after injection into BSS

### Cytotoxicity of HA derivatives

**Fig. 6:**
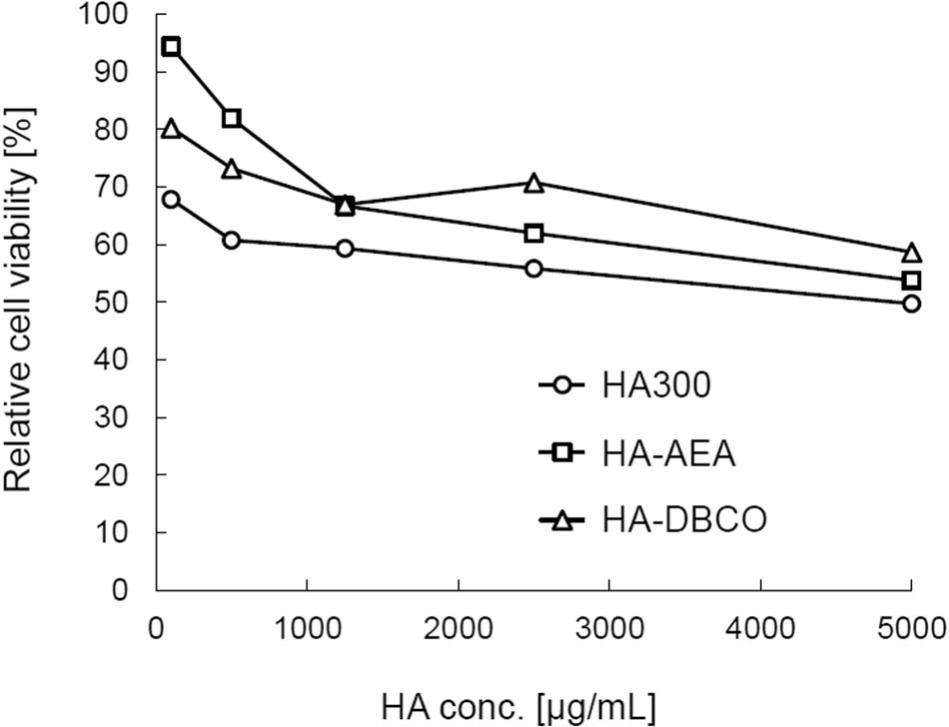
Effect of HA300, HA-AEA, or HA-DBCO on the growth of ARPE-19 cells. The effect of HA-AEA (batch 1) and HA-DBCO (batch 1) on the proliferation of ARPE-19 cells was tested in comparison with their raw material HA300. Values represent the mean ± standard deviation (*n* = 3)

ARPE-19 cells cultured in various concentrations of HA were subjected to a cell proliferation assay (Fig. 6). The relative cell proliferation was <70% in a medium containing 100 µg/mL HA and decreased depending on the concentration of HA in the medium: it was down to 49.7% in medium containing 5000 µg/mL HA. However, no abnormal cell morphological changes were observed in any culture. The results from cells grown in media containing each HA derivative were similar.

## Discussion

RVC and PM can cause secondary vitreoretinal interfacial disorders such as macular epiretinal membrane, retinal traction, or secondary retinal detachment. Surgical removal of them is one of the goals of safe and effective vitrectomy surgery. However, RVCs and PMs are hard to remove without damaging the retinal surface due to their thinness, fragility, and fibrous nature. An ideal OVD for the vitrectomy must meet several requirements. Apart from important physical properties such as optical transparency, high viscosity, and stability, it must have excellent biocompatibility due to its close contact with the retinal neuron. Furthermore, to support the resection of RVCs and PMs, it should behave like a solution until it wraps around RVCs and PMs, and then it turns to coagulation with moderate hardness.

The commercially available OVD, e.g., 1% HA-cohesive shows characteristics suitable for optical transparency, high viscosity, stability, biological compatibility, injectability, and cohesiveness. As aforementioned, some of the HA products tested could hardly be picked up with tweezers because of their weak mechanical strength. Besides, those products did not maintain their firmness and gradually spread out in aqueous solution. For developing HA-based OVDs for vitreous resection surgery, we supposed that it is essential to give them the characteristics of solidifying without spreading to BSS and other solutions. To address this issue, we sought materials that behave like a liquid after injection and solidify within a few minutes. Such materials can be expected to change into gels that can be easily picked up with tweezers while wrapping RCVs and PMs.

We have successfully developed a clinically relevant in vitro assay that likens a TA layer to the RVC (Fig. 2), the benefit of which is that TA clearly stains the vitreous and the RVC white [11, 12]. The technical practicality has been verified with Beriplast P, a commercially available fibrin glue. Beriplast P precursor solutions solidify very rapidly after mixing, even when diluted tenfold, probably because Beriplast P contains a 20–40-fold higher concentration of fibrinogen than normal blood. Therefore, Beriplast P requires optimization of the dilution rate and mixing ratio of the precursor solution to slow solidification. In this study, however, we adopted a multi-layer procedure to allow infiltration of the precursor solutions into the TA layer. Beriplast P removed TA efficiently, however, it swelled significantly in BSS. Dilution of each solution reduced swelling without affecting TA removal ability though the diluted Beriplast P showed high elasticity, which may affect its utility near the retina. As the TA removal ability of the tenfold diluted Beriplast P was 97% of the total TA (Table 2), optimization of the dilution of solutions A and B and their mixing ratio may be one approach to reaching the goal. Kaibara has reported that dynamic viscoelasticity measured by a rheometer helps to evaluate the viscosity and fluidity of blood during coagulation [35]. A commercially available hydrogel, Beriplast P was used to assess whether dynamic viscoelasticity was suitable for evaluating hydrogel properties. The G’ value of diluted Beriplast P decreased depending on its concentration, and the G’ value (e.g., fivefold dilution) increased over time, reaching 30 Pa within 5 min (data not shown). The results thus demonstrated that dynamic viscoelasticity measurements are useful for evaluating the liquidity and solidity of test materials.

An ideal adjunct to vitrectomy should have optical transparency, high viscosity, stability, and biocompatibility. Some properties have been relevant to HA products used as OVDs for cataract surgery. We assessed whether current HA products could act. The G’ was 70 Pa for 1% HA-cohesive and 15 Pa for Gel-One, suggesting that these HA products behave like solids or hydrogel under steady-state conditions. However, due to their weak biomechanical strength, they could hardly be picked up with tweezers and gradually diffused into the BSS.

Crosslinking involves the formation of bonds between polymer chains to reinforce the polymer’s structure and make it more resistant to mechanical degradation and deformation. In situ crosslinked hydrogels, in particular, are easy-to-administer aqueous injections that can reach the target site with minimal invasiveness and solidify there, suggesting the possibility of exerting functions of the ideal adjunct. We assessed whether XL-HA exhibited the physical properties of the ideal vitrectomy adjunct to vitrectomy. The DSs of HA-DBCO and HA-AEA (batch 1) were both 0.09 and the standard XL-HA prepared by mixing the 1% precursor solutions had two features: appropriate duration in the liquid state and TA removal ability (Fig. 3A and Fig. 5). The L- and S-indexes of the 1% XL-HA were 1.33 min and 3.67 min, respectively (Table 1). It has been clear that four batches of HA-DBCO and HA-AEA (batches 2–5) prepared under the standard conditions show DS and dynamic viscoelastic properties which are almost the same as those of the HA-DBCO and HA-AEA standards (batch 1), respectively. These results showed that the preparation of these HA derivatives was highly reproducible on a laboratory scale.

Figure 3B, C show the effects of precursor concentration or DS in HA derivatives on the dynamic viscoelasticity of XL-HA. Increasing concentrations of precursors shortened the L- and S-indices regardless of the difference in DS. Furthermore, when the DS increased, XL-HA showed high TA removal ability by restoring a high S-index, even at a low concentration. These results indicate that dynamic viscoelastic properties can be used not only for evaluating the liquidity and solidity of tested materials but also for quality control of HA derivatives. The 1% solutions of HA300 and standard HA derivatives dispersed rapidly in BSS (Table 2), whereas 1% HA products, 1% HA-cohesive, and Gel-One, were non-dispersive during the injection, and diffused in BSS. XL-HA showed non-dispersion and non-diffusion at a concentration above 0.76% even after standing in BSS for 5 min or longer, indicating that it had acquired strong biomechanical properties in aqueous solutions, unlike other HA products. The L-index was prolonged to more than 42 s at an XL-HA concentration of 1.30% or less. The S-index was at 3 min at an XL-HA concentration of 1.08% or more.

The effect of HA-AEA and HA-DBCO on the proliferation of ARPE-19 cells was tested in comparison with their raw material HA300 (Fig. 6). Relative cell proliferation in the presence of HA300 was reduced by 50–70%. However, we observed no morphological changes in the cells under the microscope. The decrease in cellular proliferation in the presence of HA300 suggested that the inhibition of proliferation by viscous agents such as HA occurs in an unknown mechanical manner rather than due to ordinary cytotoxicity in a biochemical manner. Cultures containing each HA derivative showed similar results. Consequently, the cell toxicity of these precursors may be comparable to that of the raw material HA300. In addition, the biocompatibility of XL-HA has been demonstrated by a subcutaneous toxicity study in mice [28].

As shown in Fig. 5, 1% XL-HA exhibited the following features: injectability via a 27‑gauge needle, non-dispersion, non-diffusion, solidification within a few minutes (enough to be picked up with tweezers), TA removal ability, and no cytotoxicity. The effect of XL-HA concentrations on TA removal ability was evaluated by the quantitative assay (Table 2). XL-HA showed TA removal ability with an efficiency exceeding 45% at a concentration of 0.76–1.73%. XL-HA improved liquidity and injectability at concentrations below 1.3%. Therefore, when HA derivatives with DS of 0.09 are used as precursors, the preferred concentration range of XL-HA is 0.97–1.30%. On the other hand, 0.78% XL-HA showed good TA removal ability when HA derivatives with DS of 0.3 are used as precursors. These results suggested that the essential factors for controlling the dynamic viscoelastic properties of XL-HA are the concentration and the DS of HA derivatives. Controlling these factors, therefore, allows us to produce XL-HA with different rheological properties, which can be used for other purposes.

Optical transparency and biological compatibility of HA-AEA and HA-DBCO as XL-HA precursors were equivalent to the starting material, HA300. As well as HA products, XL-HA showed characteristics suitable for injectability and cohesiveness. Furthermore, XL-HA, prepared in the appropriate concentration ranges, successfully embedded and removed the TA layer in vitro while exhibiting adhesion comparable to other HA products. There are still some issues, such as the long-term stability of the HA derivative of the XL-HA precursor, the performance of the animal model, and the toxic evaluation. We would like to solve these issues in the future and develop an adjunct, which leads to safe and simple vitreous surgery.

## Conclusion

HA products, including OVD for anterior segment surgery, have received tremendous support from ophthalmologists. However, applications of HA products in the posterior segment of the eye have been less successful for reasons such as a lack of biomechanical strength. In this study, by modifying HA with crosslinking technology, we have succeeded in imparting the property of solidity to HA without compromising its conventional properties. In other words, a new HA-based material gains the ability to become embedded, solidify, and be gripped in addition to a liquid. In an in vitro model in which TA mimics RVC, XL-HA reached a hardness that allowed us to lift the hydrogel with tweezers in a few minutes and thereby remove more than 45% of the total TA. These results demonstrate that XL-HA, an HA product produced via crosslinking technology, can be successfully applied in vitrectomy surgeries. Ex vivo or in vivo experimental data in anatomical eyes such as pigs are essential to evaluate the potential of XL-HA in more detail.
